# Health and Wellbeing of Older People in Mongolia: A Population-Based Survey

**DOI:** 10.1177/10105395251315885

**Published:** 2025-02-26

**Authors:** Robert G. Cumming, Gantuya Dorj, Vasoontara Sbirakos Yiengprugsawan, Jocelyn G. Dracakis, Undram Lkhagvaa, Nirmala Naidoo, Paul Kowal

**Affiliations:** 1 Australian Research Council, Centre of Excellence in Population Ageing Research (CEPAR), University of New South Wales, Kensington, NSW, Australia; 2 School of Public Health, Faculty of Medicine and Health, The University of Sydney, Sydney, NSW, Australia; 3 School of Public Health, Mongolian National University of Medical Sciences, Ulaanbaatar, Mongolia; 4 World Health Organization, Geneva, Switzerland; 5 Health Data Analytics Team, College of Health and Medicine, Australian National University, Canberra, ACT, Australia

**Keywords:** disability, Mongolia, older people, quality of life

## Abstract

The objectives of this survey were to describe the health of older Mongolians and compare those living in rural areas, urban apartment areas, and urban ger areas in the capital, Ulaanbaatar. A population-based random sample survey of 975 people aged 60 years and older was conducted in 2017 to 2018. Data were collected using methods of the World Health Organization’s Study on global AGEing and adult health. The most common chronic self-reported health conditions were hypertension (65%), arthritis (40%), and angina (23%). Most (80%) reported they were satisfied with life and the mean World Health Organization Quality of Life score on a scale of 0 to 100 was 70.6, which is relatively high. There was a higher prevalence of activities of daily living (ADLs) disability in rural areas, with 17% reporting severe difficulty with at least one ADL. High levels of disability in rural areas suggest the need for improved health and social services, including housing, for older people living outside Ulaanbaatar.

## What We Already Know

- Mongolia has a population of 3 million people, many of whom still live a nomadic lifestyle.
- Mongolia’s population is aging.
- Little is known about the health of older Mongolians.

## What This Article Adds

- Hypertension is very common in older Mongolians.
- Levels of disability are higher in rural than urban areas.
- Older Mongolians report high levels of satisfaction with life overall and also have relatively high WHOQOL scores.

## Introduction

Mongolia is the least densely populated country in the world, with 3.3 million people, of whom about a third still lead a traditional nomadic lifestyle.^1^ The World Bank currently classifies Mongolia as having a lower-middle income economy.^2^ Life expectancy in 2019 was 63.8 years for men and 72.7 years for women.^3^ The population is aging, with the percentage of the population aged 60 years and older predicted to increase from 7% in 2018 to 19% in 2050.^4^ However, there have been very few studies of the health of older Mongolians.^5,6^ In this paper, we report results of a survey of a representative sample of older Mongolians using the methodology of the World Health Organization Study on global AGEing and adult health (WHO-SAGE).^7,8^ SAGE is a study of aging and adult health in six low- and middle-income countries: China, Ghana, India, Mexico, Russia, and South Africa. Nationally representative surveys of people aged 50 years and older in these countries were conducted between 2007 and 2010. Due to budgetary constraints, the sample size in Mongolia of just under 1000 was smaller than surveys in SAGE countries (wave 1 range: 2315 in Mexico to 13 367 in China).

We designed the study so that the distribution of areas of residence of participants would be similar to Mongolia overall. About 50% of Mongolia’s population live in Ulaanbaatar, the capital and only large city.^1^ In the last 20 years, there has been significant internal migration from rural areas into Ulaanbaatar.^9^ Most of these migrants live in so-called ger districts, with poor quality housing and limited infrastructure and public services.^10,11^ It is estimated that about 50% of Ulaanbaatar’s population live in ger districts.^12^ Note that only about half the people in ger districts live in gers (yurts), the remainder live in dwellings of wood or bricks which they have built themselves.^10^

The objectives of this survey were to describe the health of Mongolians aged 60 years and older and compare those living in rural areas, urban apartment areas, and urban ger areas. A secondary objective was to compare the health of older Mongolians with the health of older people in the six countries that participated in SAGE wave 1. We have previously used our survey data to describe health service utilization among older Mongolians.^13^

## Methods

We conducted a population-based survey of the health of people in Mongolia aged 60 years and older. Data were collected using slightly shortened versions of the questionnaires developed for WHO-SAGE.^7,8^ Our survey was implemented between November 2017 and April 2018. The Research Ethics Committee of the Mongolian National University of Medical Sciences approved our research (approval: 2017/3-08) on June 9, 2017. Written consent was obtained from participants.

### Selection of Study Subjects

The survey was conducted in Ulaanbaatar (the only large city in Mongolia) and in two rural areas (aimag in Mongolian): Dundgovi and Uvurkhangai. We used a complex survey design with stratification, random sampling of clusters of people (in urban areas) and then random sampling of individual participants.

Ulaanbaatar consists of nine districts. We first randomly selected four (Bayanzurkh, Chingeltei, Khan-Uul, and Sukhbaatar) of the six central districts and one (Nailakh) of the three peripheral districts. Each district in Ulaanbaatar is divided into khoroo (between 7 and 28 in each study district) and these khoroo are classified as ger khoroo, apartment khoroo, or mixed khoroo. We randomly selected one ger khoroo and one apartment khoroo from each of the five study districts. In each of the ten study khoroo, we used lists of names held by family health centers to randomly select 50 people aged 60 years and older.

We did not have the resources to conduct a national survey across rural Mongolia. Instead, we purposively selected two rural areas (aimags) close to Ulaanbaatar: Dundgovi and Uvurkhangai. Aimag are divided into soum (15 soum in Dundgovi and 19 soum in Uvurkhangai). We purposively selected two soum from each study aimag: the soum with the largest population (Saintsagaan and Arvaikheer) and the soum with the second largest population (Erdenedalei and Kharkhorin). In each of the four study soum, we used lists of names held by the soum health center to randomly select 125 people aged 60 years and older. Each khoroo and each soum in Mongolia has just one health center and registration with the health center is mandatory for all residents.

### Data Collection

The survey team participated in a one-week training workshop led by a WHO-SAGE co–Principal Investigator (NN). Data were collected through face-to-face interviews using structured questionnaires and health measurements and took between 45 and 90 minutes to complete. Data reported in this paper include sociodemographics, life satisfaction, self-rated health, self-reported chronic health conditions, disability (including WHODAS—WHO Disability Assessment Schedule),^14^ and health-related quality of life (eight-item WHOQOL—WHO Quality of Life).^15^ Wealth tertiles were generated using an asset-based approach (possession of assets and dwelling characteristics),^16^ with tertile 1 the poorest households and tertile 3 the richest. Measured health items and performance tests were height, weight, walking speed, and grip strength.

Life satisfaction was assessed by asking, “Taking all things together, how satisfied are you with your life as a whole these days?,” with possible responses “very satisfied,” “satisfied,” “neither satisfied nor dissatisfied,” “dissatisfied,” and “very dissatisfied.” Self-rated health was assessed with the question, “In general, how would you rate your health today?,” with possible responses “very good,” “good,” “moderate,” “bad,” and “very bad.” Chronic conditions were ascertained by asking whether a health care professional/doctor had ever told them that they had: angina, arthritis, asthma, chronic lung disease, diabetes, high blood pressure (hypertension), or stroke.

Disability was assessed using WHODAS, which is based on 12 questions (each scored 1-5) assessing six domains of day-to-day functioning over the previous 30 days: cognition, mobility, self-care, getting along with people, life activities, and participation in society.^14^ Results of each item were summed and then transformed to a score between 0 and 100. We inverted the score so that high scores indicate better functioning. Three questions not included in WHODAS were also used to assess mobility, self-care, and cognition: “overall in the last 30 days, how much difficulty did you have with”: “moving around” (mobility); “self-care, such as bathing/washing or dressing yourself” (self-care), and “concentration or remembering things” (cognition). Possible responses to each question were “none,” “mild,” “moderate,” “severe,” and “extreme/cannot do.” Five basic activities of daily living (ADLs) were assessed in the same way: washing, dressing, eating, transferring (getting up from lying down), and using the toilet.

The eight-item WHOQOL was used to assess quality of life, with two questions for each of four domains: physical, psychological, social, and environmental.^15^ Results for each question (scored 1-5) were summed and transformed to a score from 0 to 100, with higher scores indicating better quality of life.

Grip strength (in kilograms) was measured twice in each hand and the mean value of the strongest hand is reported here. Gait speed (meters per second) was assessed over a 4-m course, with participants asked to walk at their normal speed. Height and weight were measured and used to calculate body mass index (BMI). Participants were categorized as underweight (BMI <18.5 kg/m^2^), normal (BMI 18.5-24.9 kg/m^2^), overweight (BMI 25-29.9 kg/m^2^), or obese (BMI ≥30 kg/m^2^).

### Sample Size and Statistical Analysis

Our target sample size was 1000 people aged 60 years and older, with 500 from Ulaanbaatar (250 from ger khoroo and 250 from apartment khoroo) and 500 from rural areas. A sample size of 1000 and an assumed design effect of 2 give 95% CIs of ±5% around point estimates of prevalence and a sample size of 500 gives 95% CIs of ±6%. With 500 in each group, the study has 80% power, with a *P* value of .05, to detect 12% differences in prevalence between urban and rural groups. With 250 in each group, we had 80% power to detect 17% differences between urban ger and apartment groups.

Data were analyzed using the Complex Samples function in SPSS Version 29 to adjust for design effects and weighting for participant sampling probability. All *P* values reported in this survey are after adjustment for age and sex using logistic regression for categorical dependent variables and linear regression for continuous variables.

## Results

A total of 975 people participated in the survey: 497 from Ulaanbaatar (*n* = 242 from urban apartment areas and *n* = 255 from urban ger areas) and 478 from the two rural areas. The participation rate was 98.3% (99.4% in Ulaanbaatar and 97.2% in the rural areas).

Participants were aged 60 to 93 years (median age: 68 years) and 61% were female (Table 1). Participants living in urban apartment areas of Ulaanbaatar tended to be older, better educated, and wealthier than those living in rural areas or urban ger areas. Participants from rural areas were the least well educated (39% with primary school or no formal education) and poorest (42% in lowest wealth tertile).

**Table 1.** Demographic and Socioeconomic Characteristics of Study Participants According to Area of Residence, Mongolia 2017 to 2018.

	Urban apartment (*n* = 242)	Urban ger (*n* = 255)	Rural (*n* = 478)	All (*n* = 975)
Female	161 (66.5%)	166 (65.1%)	268 (56.1%)	595 (61.0%)
Age (years; median)	70	66	67	68
Age group (years)				
60-69	114 (47.1%)	177 (69.4%)	299 (62.6%)	590 (60.5%)
70-79	85 (35.1%)	71 (27.8%)	137 (28.7%)	293 (30.0%)
80+	43 (17.8%)	7 (2.8%)	42 (8.8%)	92 (9.5%)
Marital status				
Married	130 (53.7%)	132 (51.8%)	305 (63.8%)	567 (57.5%)
Widowed	105 (43.4%)	115 (45.1%)	147 (30.8%)	367 (37.3%)
Not married^a^	7 (2.9%)	8 (3.1%)	26 (5.4%)	41 (4.2%)
Education^b^				
No formal	4 (1.6%)	4 (1.6%)	24 (5.1%)	32 (3.3%)
Primary school	12 (5.0%)	18 (7.2%)	159 (33.8%)	189 (19.6%)
Secondary school	46 (19.0%)	74 (29.5%)	107 (22.8%)	227 (23.6%)
High school	47 (19.4%)	39 (15.5%)	59 (12.6%)	145 (15.1%)
Tertiary	133 (55.0%)	116 (46.2%)	121 (25.7%)	370 (38.3%)
Wealth tertiles^b^				
Tertile 1 (lowest)	48 (24.9%)	68 (29.4%)	162 (41.6%)	278 (34.2%)
Tertile 2	43 (22.3%)	67 (29.0%)	154 (39.6%)	264 (32.5%)
Tertile 3 (highest)	102 (52.9%)	96 (41.6%)	73 (18.8%)	271 (33.3%)

a Never married or divorced.

b Missing data for education (n=12) and wealth (n=162).

Ten percent of participants rated their health as bad or very bad, with no difference by area of residence (Table 2). Overall, 80% were satisfied or very satisfied with life: statistically significantly lower (*P* = .04 after adjusting for age and sex) in rural areas (74%) than urban apartment (88%) or urban ger areas (84%). The most commonly reported health condition was hypertension (65%), followed by arthritis (40%) and angina (23%). There was higher prevalence of angina in urban apartment areas and lower prevalence of diabetes and hypertension in rural areas but these differences were not statistically significant (*P* > .05 after adjusting for age and sex).

**Table 2.** Health, Wellbeing, and Disability Among Study Participants According to Area of Residence, Mongolia 2017 to 2018.

	Urban apartment (*n* = 242)	Urban ger (*n* = 255)	Rural (*n* = 478)	All (*n* = 975)
Health and wellbeing				
Self-rated health				
Good, very good	72 (29.8%)	73 (28.6%)	120 (25.1%)	265 (27.2%)
Moderate	146 (60.3%)	157 (61.6%)	309 (64.6%)	612 (62.8%)
Bad, very bad	24 (9.9%)	25 (9.8%)	49 (10.3%)	98 (10.1%)
Satisfied with life^a^	213 (88.0%)	214 (83.9%)	354 (74.1%)*	781 (80.1%)
WHOQOL (mean/SD)	70.5 (10.2)	69.5 (10.2)	71.2 (13.1)	70.6 (11.7)
Chronic conditions (self-reported)				
Angina	68 (28.1%)	59 (23.1%)	98 (20.5%)	225 (23.1%)
Arthritis	113 (46.7%)	107 (42.0%)	167 (34.9%)	387 (39.7%)
Asthma	16 (6.6%)	25 (9.8%)	25 (5.2%)	66 (6.8%)
Diabetes	24 (9.9%)	23 (9.0%)	19 (4.0%)	66 (6.8%)
Hypertension	177 (73.1%)	182 (71.4%)	271 (56.7%)	630 (64.6%)
Lung disease	28 (11.6%)	36 (14.1%)	39 (8.2%)	103 (10.6%)
Stroke	26 (10.7%)	22 (8.6%)	42 (8.8%)	90 (9.2%)
Physical performance^b^				
Gait speed (m/s; mean/SD)	0.59 (0.17)	0.65 (0.24)	0.55 (0.20)	0.58 (0.21)
Grip strength (kg; mean/SD)	22.5 (8.8)	25.1 (7.3)	25.9 (10.7)	24.8 (9.6)
Body mass index^b^				
Underweight	1 (0.4%)	3 (1.2%)	7 (1.5%)	11 (1.2%)
Normal	78 (34.4%)	89 (35.9%)	172 (37.6%)	339 (36.4%)
Overweight	92 (40.5%)	107 (43.1%)	169 (37.0%)	368 (39.5%)
Obese	56 (24.7%)	49 (19.8%)	109 (23.9%)	214 (23.0%)
Disability				
Mobility disability				
None	116 (47.9%)	119 (46.7%)	222 (46.4%)	457 (46.9%)
Mild/moderate	104 (43.0%)	125 (49.0%)	217 (45.4%)	446 (45.7%)
Severe	22 (9.1%)	11 (4.3%)	39 (8.2%)	72 (7.4%)
Self-care disability				
None	182 (75.2%)	200 (78.4%)	275 (57.5%)	657 (67.4%)
Mild/moderate	48 (19.8%)	49 (19.2%)	165 (34.5%)	262 (26.9%)
Severe	12 (5.0%)	6 (2.4%)	38 (7.9%)*	56 (5.7%)
Cognition disability				
None	143 (59.1%)	153 (60.0%)	211 (44.1%)	507 (52.0%)
Mild/moderate	93 (38.4%)	93 (36.5%)	223 (46.7%)	409 (41.9%)
Severe	6 (2.5%)	9 (3.5%)	44 (9.2%)*	59 (6.1%)
ADL disability^c^				
Washing	11 (4.5%)	5 (2.0%)	48 (10.0%)**	64 (6.6%)
Dressing	6 (2.5%)	2 (0.8%)	31 (6.5%)***	39 (4.0%)
Eating	13 (5.4%)	5 (2.0%)	39 (8.2%)**	57 (5.9%)
Transferring	20 (8.3%)	11 (4.3%)	52 (10.9%)	83 (8.5%)
Toileting	11 (4.6%)	4 (1.6%)	39 (8.2%)**	54 (5.5%)
≥1 ADL disability	28 (11.6%)	17 (6.7%)	80 (16.7%)	125 (12.8%)
WHODAS (mean/SD)	85.6 (16.0)	89.5 (14.3)	78.5 (22.0)*	83.2 (19.4)

n=975. *P* values are adjusted for age and sex.

Abbreviations: ADL, activity of daily living; WHOQOL, World Health Organization Quality of Life.

a “Very satisfied” or “satisfied” with life as a whole.

b Missing data for gait speed (n=85), BMI (n=43), and grip strength (n=27).

c Severe or extreme difficulty, or unable to do the activity of daily living (ADL) task.

* *P* < .05. **P < .01. ***P < .001.

Mean gait speed was 0.58 m/s; mean grip strength was 24.8 kg; and 23% of participants were obese (Table 2). Ninety per cent of participants had a gait speed less than or equal to 0.8 m/s (data not shown). There were no differences by area of residence.

Table 2 includes measures of disability among participants in our study. Mean WHODAS scores were 89.5 in urban ger areas, 85.6 in urban apartment areas, and 78.5 in rural areas (*P* = .02 after adjusting for age and sex). A higher percentage of participants in rural areas reported severe difficulty with self-care (adjusted *P* value = .04) and cognition (concentration and remembering; adjusted *P* value = .01). Rural participants were also more likely to have ADL disability in washing (adjusted *P* value = .002), dressing (adjusted *P* value < .001), eating (adjusted *P* value = .01), and toileting (adjusted *P* value = .002).

Table 3 compares some overall health measures in those aged 70 years and older in our Mongolian survey with results from people in this age group in the six countries in the WHO-SAGE study.^7^ (WHO-SAGE study reported results for those aged 50-69 years and those 70 years and older.) The mean WHODAS score for those 70 years and older in Mongolia (76.1) was a little lower (more disability) than in China or Mexico but higher than in the other four countries. The overall mean WHOQOL score in Mongolia (69.4) was similar to Ghana, India, Russia, and South Africa but much higher than in China (52.7) and Mexico (51.5). The percent reporting they were very satisfied or satisfied with life as a whole was high in Mongolia (78%), second to Mexico (83%), with the other five WHO-SAGE countries ranging from 47% in Russia to 67% in South Africa.

**Table 3.** WHODAS (Mean) and WHOQOL (Mean) Scores and Life Satisfaction (Percentage) Among People Aged 70 Years and Older in Mongolia Survey and WHO-SAGE National Surveys in China, Ghana, India, Mexico, Russia, and South Africa.^7^

	WHODAS^a^ (median)	WHOQOL^b^ (median)	Satisfied with life^c^ (%)
Mongolia	76.1	69.4	78.1
China	83.4	52.7	62.4
Ghana	70.2	69.7	48.9
India	61.7	69.4	58.5
Mexico	79.0	51.5	83.0
Russia	67.2	68.9	46.8
South Africa	70.1	67.5	66.5

Abbreviation: WHOQOL, World Health Organization Quality of Life.

a High scores represent less disability.

b High scores represent higher quality of life.

c “Very satisfied” or “satisfied” with life as a whole.

## Discussion

This survey of a representative sample of nearly 1000 Mongolians aged 60 years and older found that older people in Mongolia tend to have high quality of life, based on scores on the eight-item WHOQOL, and high levels of satisfaction with life as a whole. The main difference in health by area of residence was that levels of disability were higher in rural than urban areas.

The prevalence of overweight and obesity in our survey of people aged 60 years and older was similar to those aged 55 to 64 years in a WHO noncommunicable diseases (NCD) risk factor surveillance study conducted in Mongolia in 2013.^17^ (Upper age limit was 64 years.) The prevalence of hypertension was a little higher in our survey (65%) than the WHO risk factor study (57%). Hypertension in our study was self-reported; in the WHO risk factor study diagnosis was based on measured blood pressure and use of antihypertensives. Prevalence of diabetes in our survey (7%) was lower than in the WHO risk factor study, where it was 13% in those aged 55 to 64 years. Diagnosis of diabetes in the WHO risk factor study was based on measured blood glucose and use of medication for diabetes.

Prevalence of disability was higher in rural than urban areas. Higher levels of disability in rural areas may be related to the challenging domestic environment in which people live in rural Mongolia. The 2020 Mongolian Census found that 66% of rural residents lived in a ger and 87% needed to collect water and bring it to their dwelling.^1^ In urban areas, 25% lived in gers and 42% needed to collect water.^1^ A 2018 survey by the International Organization for Migration found that 32% of rural residents used pit toilets and 33% did not have access to electricity in their homes, compared to less than 5% of urban residents using pit toilets and less than 5% not having electricity.^9^

Ten per cent of older Mongolians in our survey rated their health as poor (bad or very bad), with no variation across areas. This is much less than in WHO-SAGE in Ghana (35% poor health) and South Africa (23%).^18,19^ The prevalence of self-rated poor health in SAGE China was higher than in Mongolia in urban and rural areas in the eight provinces studied, except for urban Shandong (6.0% poor health).^20^ Note that participants in the six WHO-SAGE countries were aged 50 years and older, while participants in our Mongolian survey were aged 60 years and older.

The top three chronic conditions reported by participants in our study were hypertension (65%), arthritis (40%), and angina (23%). The prevalence of each of these conditions was similar to SAGE Russia.^7^ Hypertension and angina were much more frequent in Mongolia than in the other five SAGE countries (China, Ghana, India, Mexico, South Africa).^7^ The prevalence of self-reported diabetes (7%) was similar to that in the six SAGE countries.^7^

Gait speed in our survey was slower than in all SAGE countries except Russia.^21^ Mean gait speed in SAGE Russia was 0.61 m/s,^21^ similar to the 0.58 m/s in our study.

We found that Mongolians aged 70 years and older had similar levels of disability to those living in SAGE countries but they had relatively high WHOQOL scores (mean score 69) and high levels of satisfaction with life overall (78%). Among the six SAGE countries, Mexico had the highest percentage of participants reporting they were satisfied with life (83%) but the lowest mean WHOQOL score (51.5), while Ghana had the highest mean WHOQOL score (69.7) and the second lowest percentage satisfied with life (49%).^7^

A recent study of 714 Mongolians aged 18 to 65 years using the WHOQOL-BREF (24 items) found relatively low scores in the physical health domain and relatively high scores on the psychological health domain.^22^ High quality of life and life satisfaction in Mongolia are consistent with the 2023 Global State of Social Connections report.^23^ This Meta-Gallup study surveyed nationally representative samples of about 1000 people aged 15 years and over in 142 countries. Mongolia had the highest level of social connectedness among all 142 countries surveyed, with 95% reporting they were “very connected” or “fairly connected” in response to the question, “In general, how connected do you feel to people?” In SAGE countries, the percentages for feeling connected were: 81% for South Africa, 78% for Ghana, 70% for Mexico, 68% Russia, and 65% for India.^23^ China did not participate in the Meta-Gallup survey.

Our survey population was broadly representative of the older population of Mongolia in terms of age, sex, and area of residence (urban apartment, urban ger, and rural). The major limitation of our study is that rural participants were from areas close to Ulaanbaatar. We purposively chose these areas because of our limited resources. Hence, our study participants may not be representative of all older people living in rural Mongolia. They are likely to have had better access to health care, infrastructure, and other resources than those living in more remote areas and we may have found greater urban-rural differences if more remote aimag had been sampled as part of our study.

A strength of our survey is that it used largely the same questionnaire as WHO-SAGE,^7,8^ facilitating cross-national comparisons. We did not modify questions for the Mongolian context, nor did we assess the reliability, validity, psychometric properties, or cultural relevance of the data collection instruments for use in Mongolia. However, most of the WHO-SAGE items and scales (eg, WHOQOL and WHODAS) were designed to be used in cross-cultural studies. One item that might be particularly sensitive to cross-cultural differences is the single WHO-SAGE question on life satisfaction.

While we used the WHO-SAGE questionnaire, we had a different survey methodology, particularly for rural areas. WHO-SAGE surveys had much larger sample sizes and covered the whole country, ensuring nationally representative samples. They also measured clinical parameters such as blood pressure and blood sugar, whereas we relied on self-report. It is also important to note that our study was conducted nearly ten years after the surveys in the six WHO-SAGE countries and changes in health and social policy and demography over time might explain some of the observed differences.

## Conclusion

This survey found that older Mongolians have relatively high quality of life and satisfaction with life as a whole. However, levels of disability were high in rural areas, suggesting the need for improved services, including housing, for older people living outside Ulaanbaatar. Our Survey confirmed that hypertension is a major health problem in Mongolia.
